# Spinal infection caused by *Coxiella burnetii*

**DOI:** 10.1186/s12879-022-07938-7

**Published:** 2023-01-06

**Authors:** Sumin Yang, Bai Xue, Xiaowen Hu, Weidong Zhou, Minglei Zhang, Mingwei Zhao

**Affiliations:** 1 Department of Orthopedics, Qingdao Chest Hospital, No. 896 Chongqing Road, Qingdao City, Shandong Province 266043 China; 2 Qingdao Municipal Centre for Disease Control and Prevention, Qingdao Institute of Prevention Medicine, Qingdao, Shandong Province China

**Keywords:** *Coxiella burnetii*, Spinal infection, Metagenomic next-generation sequencing, Case report

## Abstract

**Background:**

Spinal infection caused by *Coxiella burnetii* is rare and difficult to diagnose. Here we reported a case of spinal infection from *Coxiella burnetii* detected by the metagenomic next-generation sequencing (mNGS).

**Case presentation:**

A 66-year-old male farmer with no medical history reported severe sharp low back pain, numbness and lower limb weakness for three years. Magnetic resonance imaging (MRI) revealed bone destruction and spinal cord compression within L1 and L2. mNGS testing showed that the inspected specimen collected from spinal lesion was detected positively for *Coxiella burnetii*. After receiving the combined treatment of antibiotic therapy and surgical intervention, the patient recovered well, and the sagittal MRI showed that vertebral edema signals disappeared and the graft of bone fused 16 months after surgery.

**Conclusion:**

The mNGS may be benefit for early diagnosis and intervention of non-specific spinal infection, and future studies should validate its effectiveness for clinical use in spinal infections. Additionally, antibiotic therapy combined with surgical intervention plays an important role on the treatment of spinal infection caused by *Coxiella burnetii*.

**Supplementary Information:**

The online version contains supplementary material available at 10.1186/s12879-022-07938-7.

## Introduction

Spinal infection is an infectious disease that could invade the vertebral body, the intervertebral disc, and/or adjacent paraspinal tissue [1]. For spinal infection, early recognition and prompt intervention are benefit for improving outcome and preventing some serious complications [2]. However, due to the low specificity of signs and a wide range of pathogens, early diagnosis for spinal infection remains an important issue and poor outcome is frequently seen [3]. *Coxiella burnetii*, as a small obligate intracellular gram-negative bacterium, is a rare pathogen that could cause spinal infection. Due to the nonspecific presentation, the delay in diagnosis for spinal infection from *Coxiella burnetii* may result in invalid intervention or even death [4]. Metagenomic next-generation sequencing (mNGS), as an emerging molecular diagnostic technique recently used in the diagnosis for unexplained infections [5, 6], may be benefit for early diagnosis of non-specific spinal infection from *Coxiella burnetii*. To the best knowledge, there have been few reports describing the spinal infection from *Coxiella burnetii* as of now [4]. Here we reported a case of spinal infection from *Coxiella burnetii* detected by mNGS.

## Case presentation

### Investigations and diagnosis

A 66-year-old male farmer with no medical history reported severe sharp low back pain, numbness and lower limbs weakness for three years. Two months later, he was admitted to a primary hospital due to an acute exacerbation of low back pain. After received two months of traditional Chinese medicine treatment, he was subsequently transferred to our tertiary referral hospital for worsening lumbar pain.

**Fig. 1:**
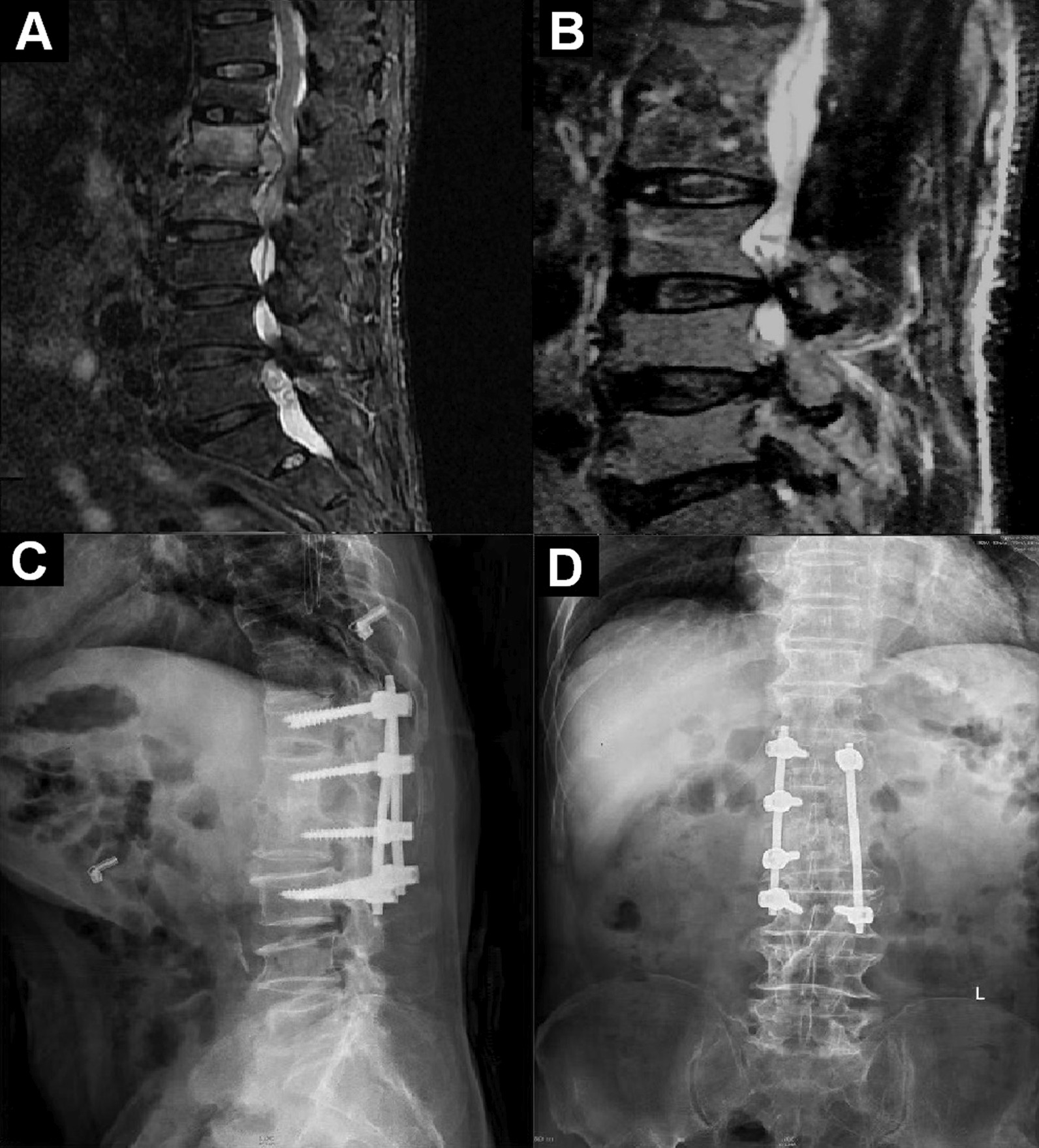
Magnetic resonance imaging (MRI) and radiograph of the patient before and after surgery (**A** T2-weighted imaging of the patient before surgery; **B** T2-weighted imaging of the patient 16 months after surgery; **C** Lateral radiograph of the spine 16 months after surgery; **D** Anteroposterior radiograph of the spine 16 months after surgery)

**Fig. 2:**
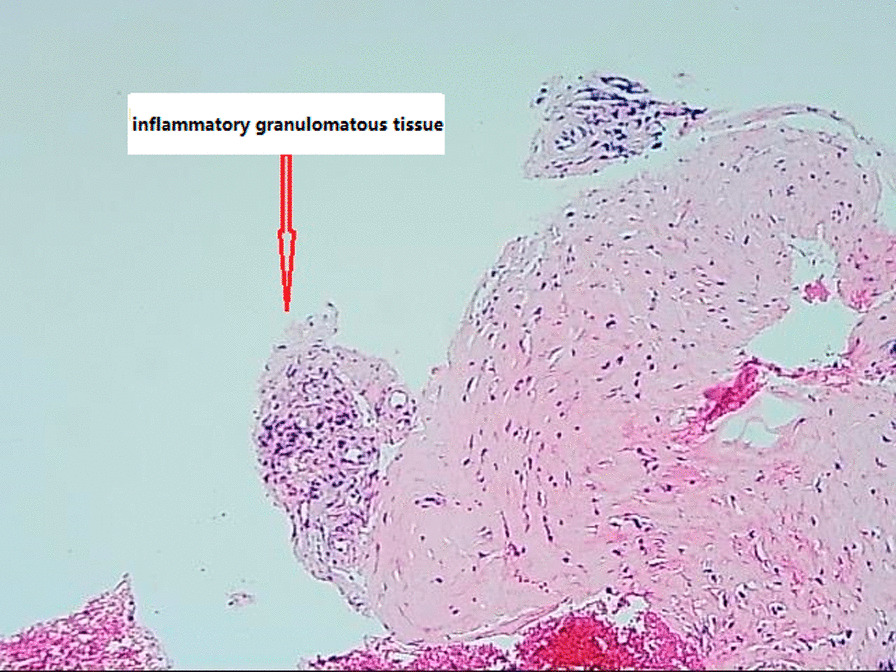
H&E Photomicrograph view (×100) showing inflammatory granulomata (red arrow) from the tissue within the intervertebral of L1 and L2 before drug treatment. Micrographic imaging was performed using an Olympus BX43 light microscope (Tokyo, Japan) furnished with a Smart V350D digital camera (JEDA Science and Technology Development Co., Ltd, Jiangsu, China) for observation and capturing respectively. Acquisition software was TianMin SDK-2000 system

In the tertiary hospital, his physical and neurologic examination was normal except for lumbar pain, with no risk factors for *Mycobacterium tuberculosis* infection. Magnetic resonance imaging (MRI) revealed bone destruction and spinal cord compression within L1 and L2 (Fig. 1). Laboratory tests were unremarkable, including erythrocyte sedimentation rate (ESR) of 9.9 mm/h (normal < 15mm/h) and C-reactive protein (CRP) of 1.05 mg/l (normal < 10 mg/l). Multiple blood cultures and serological tests for *Mycobacterium tuberculosis, Coxiella burnetii* and Brucellosis were negative as well as tuberculin testing. A CT-guided puncture biopsy from the vertebra L1-L2 yielded inflammatory granuloma (Fig. 2; Additional file 1: Fig. S1).

In order to diagnose this unexplained infection, the biopsy tissue was subjected to mNGS testing, involving in a wide range of genomic sequences for 6350 kinds of bacteria (including mycobacteria, chlamydia and rickettsia), 1798 kinds of virus, 1064 kinds of fungus and 234 kinds of parasite. Results of the mNGS testing revealed that only *Coxiella burnetii* was detected positively for the inspected specimen collected from spinal lesions. Incorporating microbiological, clinical and radiological presentations, the patient was primarily confirmed for spinal infection caused by *Coxiella burnetii* and then the therapeutic schedule was made.

### Treatment

According to the guidelines [7], therapy with rifampin (0.6g orally daily), doxycycline (0.1g orally twice a day) and levofloxacin (0.4g orally daily) were initiated and continued for 18 months at least. Inflammation was partially absorbed after one month of conservative treatment, but the extent of the lesion was not alleviated as well as the severity of symptoms (Additional file 1: Figs. S2, S3). The low back pain progressed to the point, and he was no longer able to ambulate. So the surgical debridement was recommended. After informed consent was obtained from the patient, anterior debridement and fusion plus posterior fusion with instrumentation was performed to remove eroded bone and infected associated granulomatous tissue for decompression of spinal nerve.

### Outcome and follow-up

His physical examination remained well without any signs of infection, so he was discharged to home. After 16 months of follow-up after surgery, the sagittal MRI showed that vertebral edema signals disappeared and the graft of bone fused (Fig. 1).

## Discussion and conclusion

Infections from *Coxiella burneti*i, resulting in Q fever, are relatively rare and difficult to diagnose [7]. Farmer in frequent contact with animals is a high risk group for Q fever by inhaling contaminated aerosols with pathogen, which commonly manifests self-limiting flu-like illness, pneumonia and hepatitis [8]. Chronic Q fever may develop many months to years after initial infection. Spinal infection in chronic Q fever is much rare especially for isolated spinal infection without history of vascular disease and intervention [9]. As of now, only eleven cases of isolated spinal infection from *Coxiella burnetii* except for us have been reported from a recent literature review spanning 1990–2020 performed by Ghanem-Zoubi et al. [10]. Both our case report and all other studies suggest that non-specific symptom of this rare infection makes it a diagnostic challenge, and the duration of initial symptoms until diagnosis is long varying from several months up to few years in general that often resulting in aggravation for misdiagnosis and delayed treatment [11, 12].

The diagnosis of chronic Q fever currently relies on serology and detection of DNA in blood or tissue [13]. According to the Dutch consensus guideline on chronic Q fever diagnostics, positive polymerase chain reaction (PCR) or positive serology could prove chronic infection from *Coxiella burneti*i [14]. Our case presented negative results of multiple serological tests and blood cultures for *Coxiella burnetii, Mycobacterium tuberculosis* and Brucellosis as well as tuberculin testing, but positive of the molecular test. This discrepancy between serological and molecular detection of pathogens is possible in acute infection, whereas it is unusual in chronic spinal infection which takes a considerable amount of time to produce infection in patients contact with infectious microorganism. There is no clear explanation for the discrepancy between serological and molecular detection in our clinical report. The negative serological testing is considered to be the result of a lack of sensitivity of the testing, possible due to an antigenic difference between the strain used for the test and the strain infecting the case. Moreover, the cut-off point is a methodological problem in serological testing, and chronic infection from *Coxiella burnetii* cannot be excluded when detecting negative serology, due to the still non-consensus for the cut-off value [14]. Additionally, inter and intra laboratory variations of immunofluorescence methods and results are probably of significant importance, which affects the accuracy of testing even causing false result [14]. Similarly, several reports documented the discrepancy of negative serology but positive molecular detection such as PCR, which revealed that this was common in some specific cases, however, the explanation was not described [10, 15]. In our case, a CT-guided puncture biopsy from the vertebra L1–L2 yielded inflammatory granuloma. In order to diagnose this unexplained infection, we performed mNGS for biopsy tissue after obtaining the patient’s consent. Results of the mNGS testing revealed that only *Coxiella burnetii* was detected positively for the inspected specimen collected from spinal lesion, which directly prompted the confirmation for spinal infection caused by *Coxiella burnetii* incorporating other presentation. As the development of molecular diagnosis, mNGS platforms permit rapid pathogen detection and species identification from a single clinical sample after general testing with no definite pathogens [16]. Through high-throughput sequencing and automated bioinformatics analysis, some known microbial genomes can be theoretically identified from clinical samples in a short time. In the field of infectious diseases, it is successfully used to detect pathogens in various clinical samples, such as plasma and bronchoalveolar lavage fluid [17, 18]. Recent research demonstrated that mNGS had a greater rate of pathogen detection in osteoarticular infection than that of conventional culture-based methods [19]. Our case report presents that the mNGS assay may be benefit for early identification and intervention of non-specific spinal infection, which may be a rapid and accurate way to address misdiagnosis and delayed treatment for this rare infection. However, it should be noted that not all the vertebral biopsies of the patient are subjected to mNGS assay. For non-specific spinal infection, serological test is primarily selected to identify conventional pathogens, and the serological result should be confirmed by the real-time polymerase chain reaction (RT-PCR) or by culture of the lesion. If all results are negative and the infection is still unexplained, mNGS should be recommended for rapid and accurate detection of pathogens. To date, the mNGS has not been validated for clinical use in spinal infections, and a false positive result is quite conceivable. Molecular contamination, inappropriate primers, and other factors can affect results. The conventional PCR could be subsequently requested to confirm or exclude the mNGS result. Therefore, the diagnosis of spinal infection from *Coxiella burnetii* is proposed for combination of microbiological, clinical and radiological presentations.

Although the optimal duration of therapy is unknown, the treatment of chronic Q fever is recommended to giving doxycycline at least 18 month [7]. The addition of rifampicin has also been suggested for its property of bone penetration [20]. Conservative treatment is reasonable in early stages with no or minor neurologic deficits of spinal infection. However, surgical treatment is mandatory if patients present with abscess and nerve compression, neurologic deficits, and/or progressive instability or deformity [1]. For our case, we performed a joint therapeutic regimen combining with neurosurgical intervention and long-term of antibiotic therapy. Despite the limited data available about therapeutic strategies for spinal infection caused by *Coxiella burnetii*, we consider that surgical remove of damaged bone and granulomatous tissue may be essential because of extensive lesions in the spinal infection. Additionally, long-term antibiotic therapy and regular review are very important for preventing recurrence of this disease [21].

Spinal infection from *Coxiella burnetii* is rare and difficult to diagnose. The mNGS may be benefit for early diagnosis and intervention of non-specific spinal infection, and future studies should validate its effectiveness for clinical use in spinal infections. Additionally, antibiotic treatment joint with surgical intervention plays an important role on the treatment of chronic spinal infection caused by *Coxiella burnetii*.

## Supplementary information

**Additional file 1**: **Figure S1** H&E Photomicrograph view (×400) showinginflammatory cell infiltration (redarrow) from the tissue within the intervertebral of L1 and L2 before drugtreatment. Micrographic imaging was performed using an Olympus BX43 lightmicroscope (Tokyo, Japan) furnished with a Smart V350D digital camera (JEDAScience and Technology Development Co., Ltd, Jiangsu, China) for observationand capturing respectively. Acquisition software was TianMinSDK-2000 system.** Figure S2** H&E Photomicrograph view (×100) showing smallfocal inflammatory granulomata (red arrow) from the tissue within theintervertebral of L1 and L2 after a month of drug treatment. Micrographicimaging was performed using an Olympus BX43 light microscope (Tokyo, Japan)furnished with a Smart V350D digital camera (JEDA Science and TechnologyDevelopment Co., Ltd, Jiangsu, China) for observation and capturingrespectively. Acquisition software was TianMin SDK-2000 system. **Figure S3** H&E Photomicrograph view (×400) showinginflammation absorbs calcification (red arrow) from the tissue within theintervertebral of L1 and L2 after a month of drug treatment. Micrographicimaging was performed using an Olympus BX43 light microscope (Tokyo, Japan)furnished with a Smart V350D digital camera (JEDA Science and TechnologyDevelopment Co., Ltd, Jiangsu, China) for observation and capturingrespectively. Acquisition software was TianMin SDK-2000 system.

## Data Availability

Next generation sequencing data have been deposited to NCBI’s sequence read archive (SRA) under BioProject accession number PRJNA909599 (https://www.ncbi.nlm.nih.gov/bioproject/PRJNA909599).

## Abbreviations

MRI: Magnetic resonance imaging
mNGS: Next-generation sequencing
ESR: Erythrocyte sedimentation rate
CRP: C-reactive protein
