# Supplementary material for: Spinal infection caused by Coxiella burnetii
Source: BMC Infect Dis. 2023 Jan 6;23:6. doi: 10.1186/s12879-022-07938-7 (PMC9817394; doi:10.1186/s12879-022-07938-7)
Supplement: Supplementary file 1 — Additional file 1: Figure S1 H&E Photomicrograph view (×400) showinginflammatory cell infiltration (redarrow) from the tissue within the intervertebral of L1 and L2 before drugtreatment. Micrographic imaging was performed using an Olympus BX43 lightmicroscope (Tokyo, Japan) furnished with a Smart V350D digital camera (JEDAScience and Technology Development Co., Ltd, Jiangsu, China) for observationand capturing respectively. Acquisition software was TianMinSDK-2000 system. Figure S2 H&E Photomicrograph view (×100) showing smallfocal inflammatory granulomata (red arrow) from the tissue within theintervertebral of L1 and L2 after a month of drug treatment. Micrographicimaging was performed using an Olympus BX43 light microscope (Tokyo, Japan)furnished with a Smart V350D digital camera (JEDA Science and TechnologyDevelopment Co., Ltd, Jiangsu, China) for observation and capturingrespectively. Acquisition software was TianMin SDK-2000 system. Figure S3 H&E Photomicrograph view (×400) showinginflammation absorbs calcification (red arrow) from the tissue within theintervertebral of L1 and L2 after a month of drug treatment. Micrographicimaging was performed using an Olympus BX43 light microscope (Tokyo, Japan)furnished with a Smart V350D digital camera (JEDA Science and TechnologyDevelopment Co., Ltd, Jiangsu, China) for observation and capturingrespectively. Acquisition software was TianMin SDK-2000 system. [file 12879_2022_7938_MOESM1_ESM.pptx]

## Slide 1
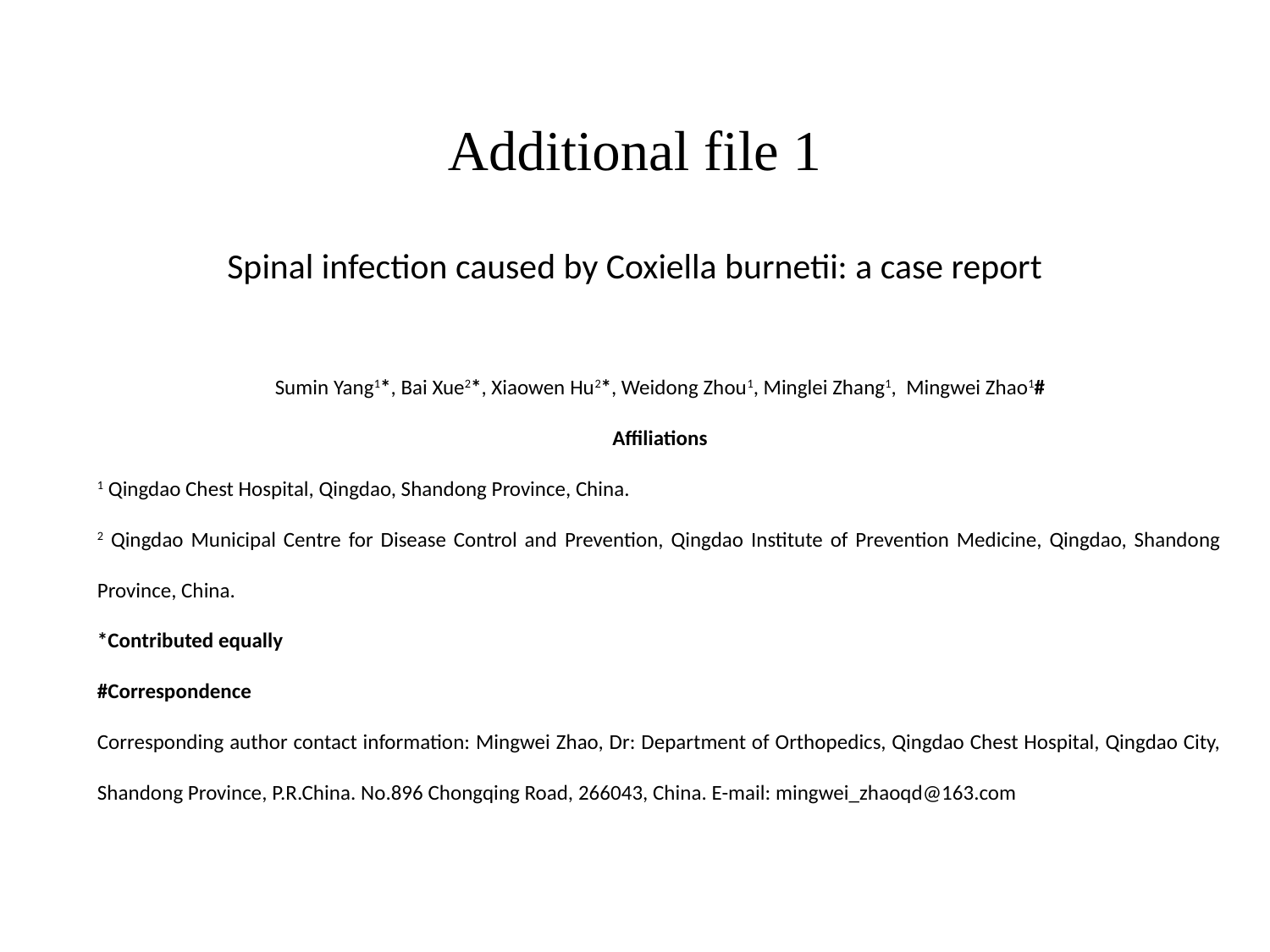

Additional file 1
Spinal infection caused by Coxiella burnetii: a case report
Sumin Yang1*, Bai Xue2*, Xiaowen Hu2*, Weidong Zhou1, Minglei Zhang1, Mingwei Zhao1#
Affiliations
1 Qingdao Chest Hospital, Qingdao, Shandong Province, China.
2 Qingdao Municipal Centre for Disease Control and Prevention, Qingdao Institute of Prevention Medicine, Qingdao, Shandong Province, China.
*Contributed equally
#Correspondence
Corresponding author contact information: Mingwei Zhao, Dr: Department of Orthopedics, Qingdao Chest Hospital, Qingdao City, Shandong Province, P.R.China. No.896 Chongqing Road, 266043, China. E-mail: mingwei_zhaoqd@163.com

## Slide 2
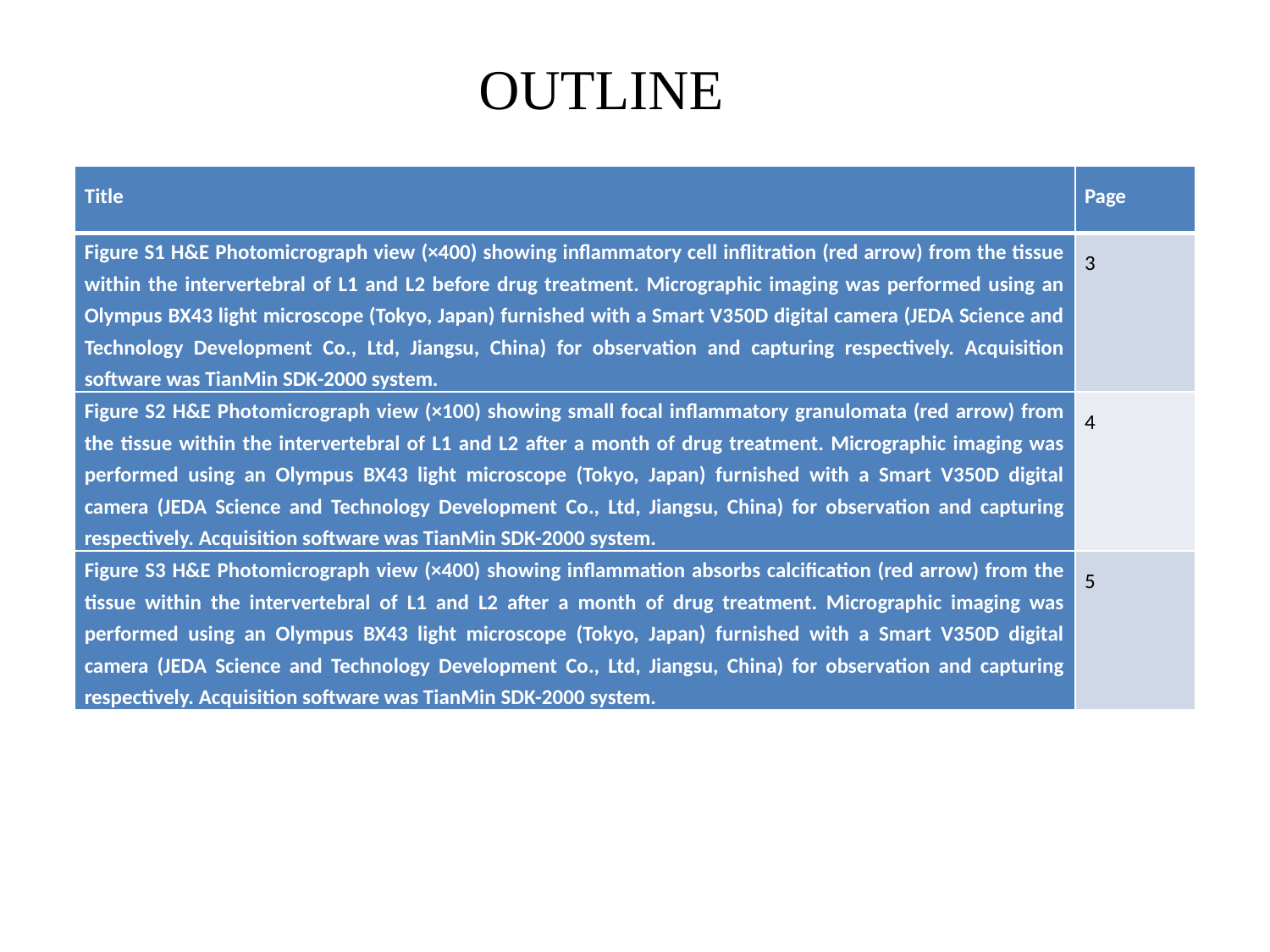

OUTLINE
| Title | Page |
| --- | --- |
| Figure S1 H&E Photomicrograph view (×400) showing inflammatory cell inflitration (red arrow) from the tissue within the intervertebral of L1 and L2 before drug treatment. Micrographic imaging was performed using an Olympus BX43 light microscope (Tokyo, Japan) furnished with a Smart V350D digital camera (JEDA Science and Technology Development Co., Ltd, Jiangsu, China) for observation and capturing respectively. Acquisition software was TianMin SDK-2000 system. | 3 |
| Figure S2 H&E Photomicrograph view (×100) showing small focal inflammatory granulomata (red arrow) from the tissue within the intervertebral of L1 and L2 after a month of drug treatment. Micrographic imaging was performed using an Olympus BX43 light microscope (Tokyo, Japan) furnished with a Smart V350D digital camera (JEDA Science and Technology Development Co., Ltd, Jiangsu, China) for observation and capturing respectively. Acquisition software was TianMin SDK-2000 system. | 4 |
| Figure S3 H&E Photomicrograph view (×400) showing inflammation absorbs calcification (red arrow) from the tissue within the intervertebral of L1 and L2 after a month of drug treatment. Micrographic imaging was performed using an Olympus BX43 light microscope (Tokyo, Japan) furnished with a Smart V350D digital camera (JEDA Science and Technology Development Co., Ltd, Jiangsu, China) for observation and capturing respectively. Acquisition software was TianMin SDK-2000 system. | 5 |

## Slide 3
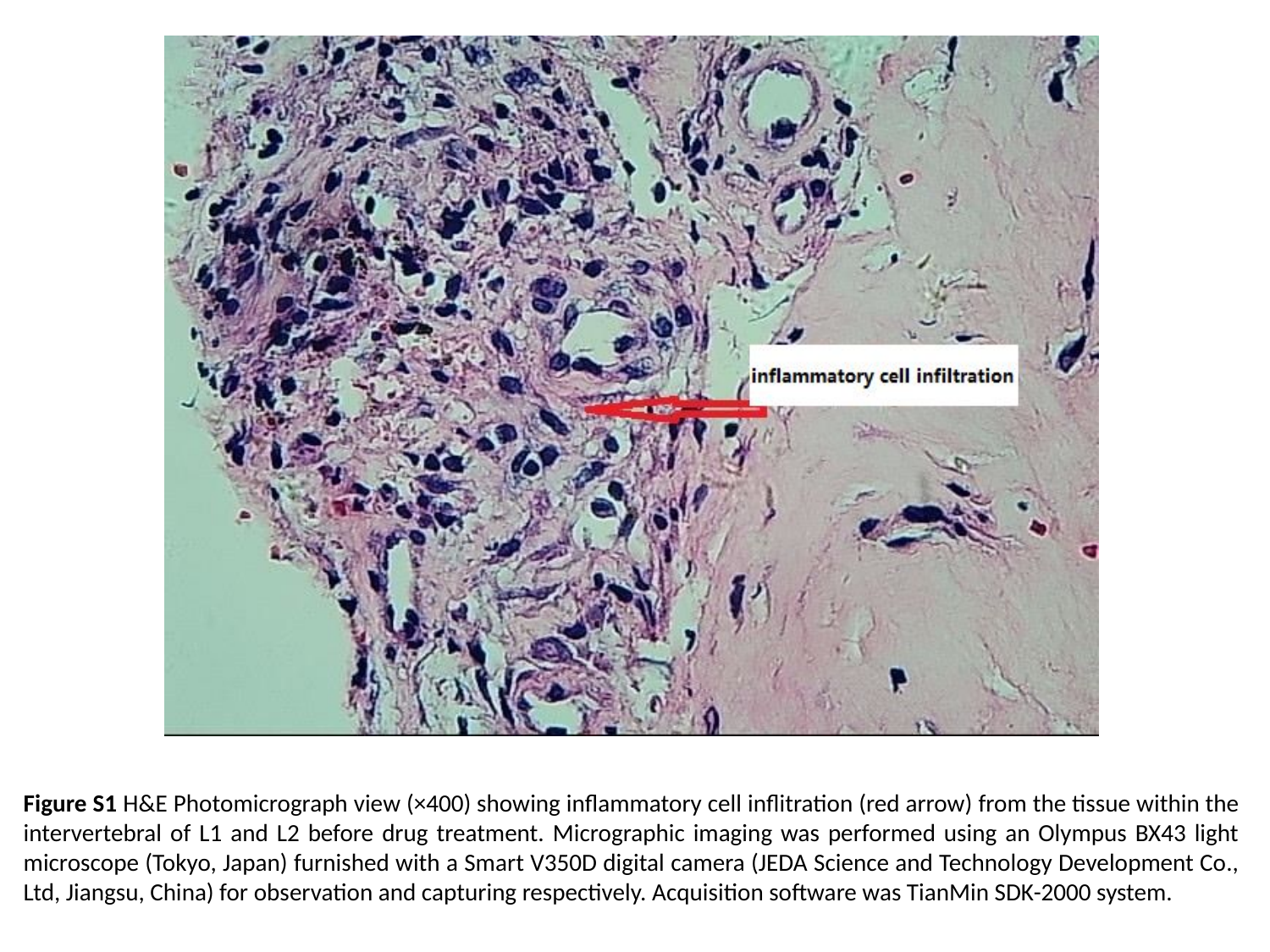

Figure S1 H&E Photomicrograph view (×400) showing inflammatory cell inflitration (red arrow) from the tissue within the intervertebral of L1 and L2 before drug treatment. Micrographic imaging was performed using an Olympus BX43 light microscope (Tokyo, Japan) furnished with a Smart V350D digital camera (JEDA Science and Technology Development Co., Ltd, Jiangsu, China) for observation and capturing respectively. Acquisition software was TianMin SDK-2000 system.

## Slide 4
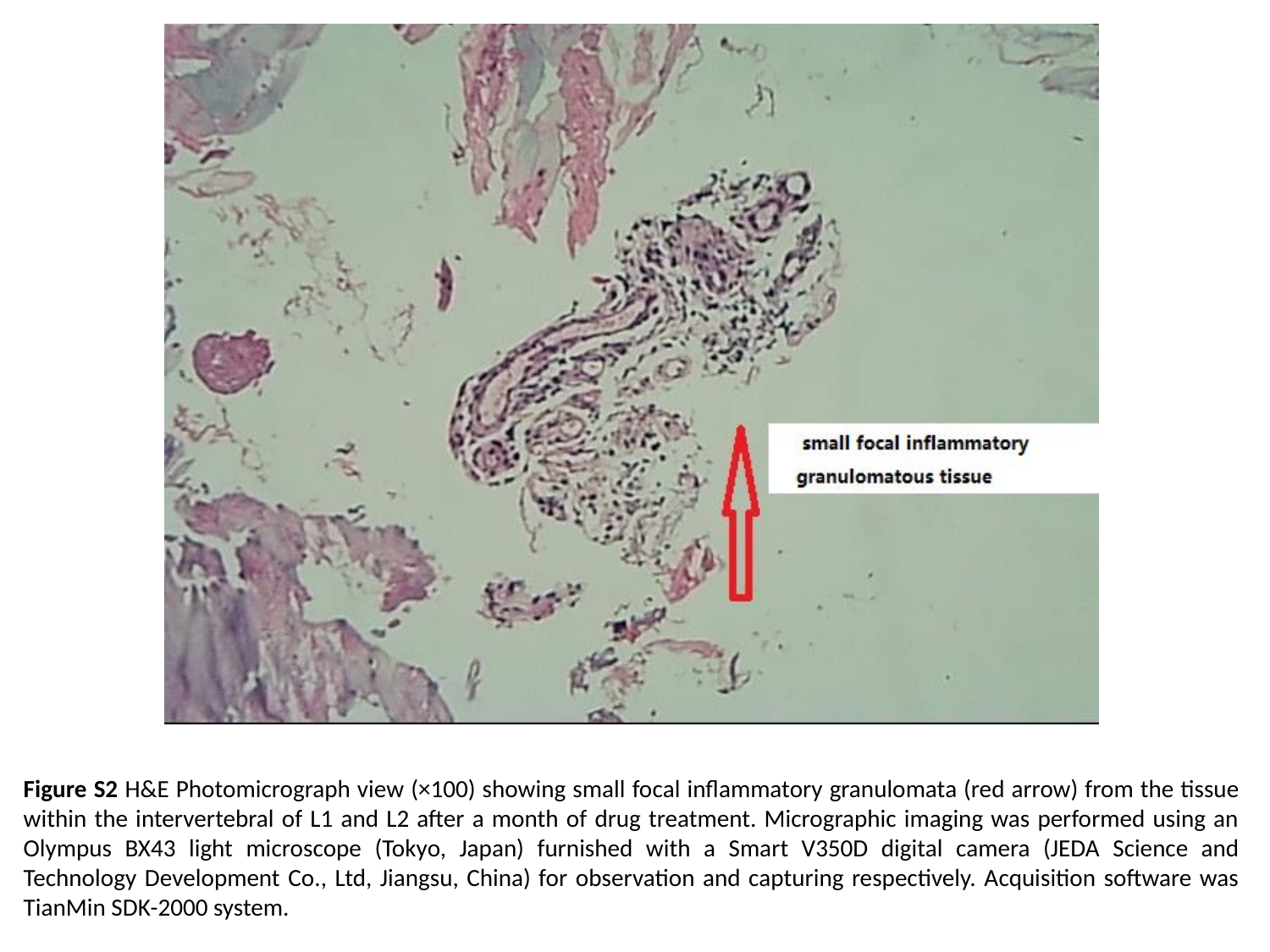

Figure S2 H&E Photomicrograph view (×100) showing small focal inflammatory granulomata (red arrow) from the tissue within the intervertebral of L1 and L2 after a month of drug treatment. Micrographic imaging was performed using an Olympus BX43 light microscope (Tokyo, Japan) furnished with a Smart V350D digital camera (JEDA Science and Technology Development Co., Ltd, Jiangsu, China) for observation and capturing respectively. Acquisition software was TianMin SDK-2000 system.

## Slide 5
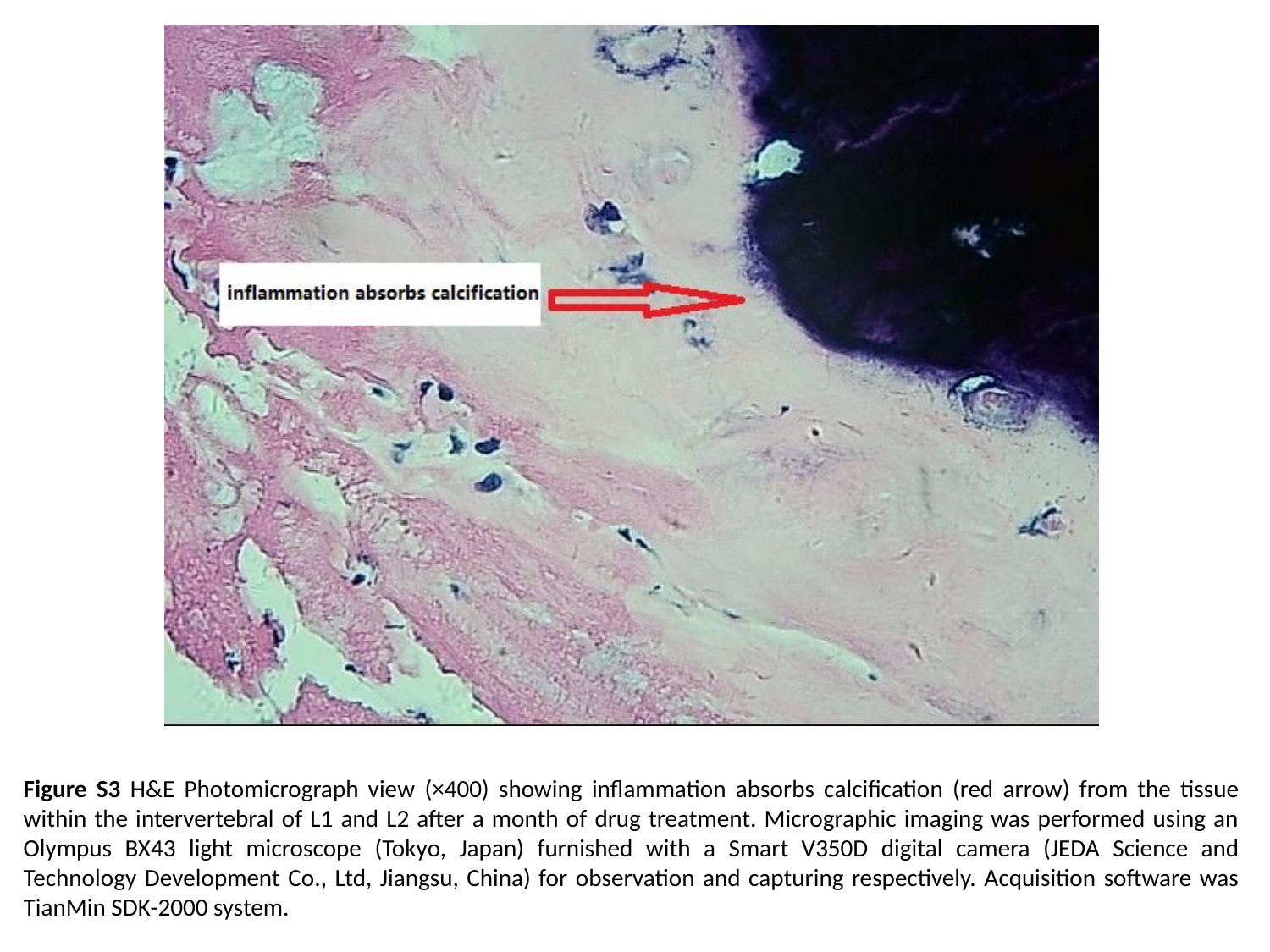

Figure S3 H&E Photomicrograph view (×400) showing inflammation absorbs calcification (red arrow) from the tissue within the intervertebral of L1 and L2 after a month of drug treatment. Micrographic imaging was performed using an Olympus BX43 light microscope (Tokyo, Japan) furnished with a Smart V350D digital camera (JEDA Science and Technology Development Co., Ltd, Jiangsu, China) for observation and capturing respectively. Acquisition software was TianMin SDK-2000 system.
